# Critical supply chains for mitigating PM_2.5_ emission-related mortalities in India

**DOI:** 10.1038/s41598-021-91438-2

**Published:** 2021-06-07

**Authors:** Haruka Mitoma, Fumiya Nagashima, Shigemi Kagawa, Keisuke Nansai

**Affiliations:** 1 Graduate School of Economics, Kyushu University, Fukuoka, Japan; 2 Faculty of Economics, Kindai University, Osaka, Japan; 3 Faculty of Economics, Kyushu University, Fukuoka, Japan; 4 National Institute for Environmental Studies, Ibaraki, Japan; 5 School of Physics, ISA, The University of Sydney, Sydney, NSW Australia

**Keywords:** Ecology, Environmental social sciences

## Abstract

Air pollution and its health-related effects are a major concern globally, and many people die from air pollution-related diseases each year. This study employed a structural path analysis combined with a health impact inventory database analysis to estimate the number of consumption-based PM_2.5_ emission-related deaths attributed to India’s power supply sector. We identified critical supply chain paths for direct (production) electricity use and indirect (consumption) use. We also considered both domestic and foreign final demand and its effect on PM_2.5_ emission-related deaths. Several conclusions could be drawn from our results. First, the effect of indirect electricity usage on PM_2.5_ emission-related deaths is approximately four times larger than that for direct usage. Second, a large percentage of pollution-related deaths can be attributed to India’s domestic final demand usage; however, electricity usage in the intermediate and final demand sectors is inextricably linked. Third, foreign final demand sectors from the Middle East, the USA, and China contribute indirectly toward PM_2.5_ emission-related deaths, specifically in the rice export supply chain. The results show that the Indian government should implement urgent measures to curb electricity use in rice supply chains in order to reduce the number of PM_2.5_ emission-related deaths.

## Introduction

A study on the global burden of disease conducted by the Institute for Health Metrics and Evaluation (IHME) showed that air pollution is the fifth highest risk factor for mortality worldwide and the leading environmental risk factor; air pollution is responsible for 4.2 million deaths annually^1,2^. Among various air pollutants, fine particulate matter measuring 2.5 µm or less in aerodynamic diameter (PM_2.5_) is sufficiently small to penetrate the lungs deeply and pass into the blood stream. This may cause cardiovascular and respiratory diseases, such as lower respiratory infection (LRI), ischemic heart disease, cerebrovascular disease, chronic obstructive pulmonary disease (COPD), and lung cancer^1–3^.

During the period 2000–2015, when the annual GDP growth rate in India exceeded 8%^4^, the number of premature deaths attributable to PM_2.5_ exposure increased from 857,300 to 1,090,400 people^1^. In 2015, PM_2.5_-related premature deaths in India accounted for a quarter of global deaths attributed to PM_2.5_, a level that was comparable to that of China, which has some of the world’s highest air pollution levels^1^.

India’s rapid economic growth between 1995 and 2009 was mainly due to increasing fixed capital formation (i.e., final demand), and the additional capital formation (i.e., investment) was attributed to a marked increase in coal consumption in India during the same period; coal consumption is one of the major sources of PM_2.5_ emissions^5^. Thus, to reduce premature deaths related to PM_2.5_ emissions in India, it is considered important for Indian policymakers to develop effective demand- and supply-side policy with a focus on higher priority sectors.

In 2019, the Indian government launched the National Clean Air Programme (NCAP) to achieve its sustainable development goals; the proposed national target was a 20–30% reduction in PM_2.5_ and PM_10_ levels by 2024^6^. This is the first time-bound commitment concerning air pollution that has been promulgated in India. Although the NCAP mentioned the importance of adopting a multi-sectoral and collaborative approach^6^, concrete collaborative policies have not yet been developed. To develop effective demand- and supply-side policies, it is important to obtain a deeper understanding of the supply chain structure centered around a critical sector that has contributed to PM_2.5_ emissions—and therefore, premature deaths—in India.

According to the Regional Emission Inventory in Asia (REAS) database for emissions from 2000 to 2008^7^, the power generation sector is one of the largest contributors of PM_2.5_ emissions in India, accounting for 822,000 tons of PM_2.5_ in 2008. In addition, the emissions from the power generation sector increased consistently from 2000 to 2008. Considering energy sources for electrical power generation in India, coal-fired thermal power accounted for 68% of the total 462 TWh generated in 2007^8^. However, coal-fired thermal power plants were responsible for more than 90% of PM_2.5_ emissions in the power generation sector in 2007^7^, which means that coal-fired thermal power is the most emission-intensive sector and that it plays a critical role in the emissions-related health impact on the people of India. This study examined power generation sector including the coal-fired thermal power and oil-fired thermal power generation, biomass power generation, which account for the remaining 10% of PM_2.5_ emissions as a critical emission source sector.

PM_2.5_ emissions from the electric power sector have been increasing due to the increases in electric power consumption that is *directly* necessary for households, and for industries that produce “final” goods and services. In addition to direct electric power use, it is also important to note that both consumers, i.e., households and industry, also *indirectly* consume electric power through the production of “intermediate” goods and services (including electric power) that are required to produce the final goods and services. It is also important to note that both direct and indirect electric power consumption generate PM_2.5_ emissions.

The electric power generation sector plays an important role in the supply chain^9^. To effectively mitigate the health impacts related to PM_2.5_ emissions in India, the PM_2.5_ emissions associated with the indirect use of electricity (i.e., Scope 3 emissions from the electricity sector in line with the greenhouse gas [GHG] protocol^10^, as well as emissions associated with the direct use of electricity (i.e., Scope 2 emissions from the electricity sector in line with the GHG protocol^11^) need to be reduced. In other words, it is necessary to identify environmentally important supply chain paths that have the greatest mitigation potential for health impacts in India.

A highly relevant study by Guttikunda and Jawahar (2014)^12^ focused on coal-fired power plants located in Indian states in 2010 and estimated the total annual PM_2.5_ emissions in India at around 580,000 tons. These authors also estimated that the annual PM_2.5_-induced mortalities in India were between 80,000 and 115,000. However, because the study of Guttikunda and Jawahar (2014)^12^ only examined “production-based” PM_2.5_ emissions and production-based mortality risks, these results provide a relatively limited understanding of how the final demand of countries such India affects PM_2.5_-induced mortality risks.

Nansai et al. (2020)^13^ quantified the mortality-based economic losses (i.e., income loss) attributed to primary and secondary PM_2.5_ emissions in individual Asian countries that were induced by the final demand of the world’s five largest consuming countries. Their findings showed that in 2010, consumption in the USA, China, Japan, Germany, and the United Kingdom caused approximately 2000, 7700, 2700, 3300, and 3400 deaths in India, respectively. These deaths resulted in economic losses in India of 0.14, 0.26, 0.087, 0.11, and 0.11 billion US dollars in purchasing power parity, respectively. In India, particularly, the export of goods and services from India to these developed countries contributed considerably to PM_2.5_ emissions, and therefore the high number of premature deaths in India. This situation calls for an analysis of how the global supply chain is impacting health in India in terms of emission responsibility^14^. In addition, domestic policies need to be introduced to mitigate air pollution inside India, and demand-side policies that consider the role of consumers outside India need to be developed.

Structural path analysis (SPA) is a well-known and effective method that was first introduced by Defourny and Thorbecke (1984)^15^ to trace important supply chain paths from complex input–output structures by decomposing matrix products into elements (paths). Previous studies addressing PM_2.5_ emissions have applied this method. For example, Meng et al. (2015)^16^ identified PM_2.5_ emission-intensive supply chain paths in China using SPA. However, they only considered PM_2.5_ emissions and did not consider the reduction potential of health impacts. Nagashima et al. (2017)^17^ identified critical supply chain paths that contribute toward premature deaths in East Asian countries; however, they did not include secondary PM_2.5_ generation, which has a marked influence on health, and they did not consider India in their analysis.

This study used EXIOBASE 3 data for 2010 and applied an SPA^18–21^ to identify important supply chain paths driven by domestic and international demands that contribute to primary and secondary PM_2.5_ emissions from the power sector, which is an environmentally critical sector in India. We introduced an atmospheric transport model to fully link final demand via supply chains to the primary emitter that is the power sector in India. Finally, we linked the atmospheric transport of emissions from the emitter to the impact on health in India. To the best of our knowledge, this study is the first attempt to estimate consumption-based PM_2.5_ emissions as well as the consumption-based mortality risk in India by using a combined approach that is based on an environmentally extended multi-regional input–output (MRIO) analysis and an atmospheric transport model.

The remainder of this manuscript is structured as follows: “Methodology” section explains our methodology, “Data and computation” section describes the data, “Results” section presents and discusses the results, and finally, “Discussion and conclusion” section contains the discussion and conclusions.

## Methodology

To estimate consumption-based PM_2.5_ emissions, we employed the consumption-based health impact accounting method developed by Takahashi et al. (2014)^22^ and Nagashima et al. (2017)^17^.

### Consumption-based PM_2.5_ emissions

The output vector, ${\varvec{x}}=\left({x}_{i}^{r}\right)$ (*r* = 1,…,*R*, *i* = 1,…, *N*) in a MRIO analysis with *N* industries and *R* countries can be obtained using the equation ${\varvec{x}}={\varvec{A}}{\varvec{x}}+{\varvec{f}}$; where ${{\varvec{f}}}^{g}=\left({f}_{i}^{rg}\right)$ is the global final demand vector that shows the final demand for goods and services flowing from industry *i* in country *r* to the final consumers of *R* countries; ${\varvec{A}}=\left({Z}_{ij}^{rs}/{x}_{j}^{s}\right)=\left({a}_{ij}^{rs}\right)$ is an input coefficient matrix showing the intermediate input of industry *i* in country *r* that is necessary for producing one unit of the product from industry *j* in country *s*, for which ${Z}_{ij}^{rs}$ represents the intermediate input into industry *j* of country *s* from industry *i* of country *r*.

The global final demand vector, ${{\varvec{f}}}^{g}$, can be expressed as the sum of the final demand vectors of each demand country: ${{\varvec{f}}}^{g}={{\varvec{f}}}^{1}+{{\varvec{f}}}^{2}+\dots +{{\varvec{f}}}^{R}=\sum_{s=1}^{R}{{\varvec{f}}}^{s}$; where, ${{\varvec{f}}}^{s}$ is the domestic final demand vector for country *s*. Solving the above equation for the output vector ***x*** yields the following:

1 $${\varvec{x}}={\left({\varvec{I}}-{\varvec{A}}\right)}^{-1}{\varvec{f}}={\varvec{L}}{{\varvec{f}}}^{{\varvec{g}}}$$

where ***I*** is the identity matrix and ${\varvec{L}}={({\varvec{I}}-{\varvec{A}})}^{-1}=\left({L}_{ij}^{rs}\right)$ is the Leontief inverse matrix with elements ${L}_{ij}^{rs}$ expressing the outputs of industry *i* in country *r* that are directly and indirectly required to satisfy one unit of final demand from industry *j* in country *s*.

The electricity output in India triggered by the final demand in country *s* can be estimated as follows:

2 $${e}^{s}={{\varvec{w}}{^{\prime}}\left({\varvec{I}}-{\varvec{A}}\right)}^{-1}{{\varvec{f}}}^{s}={\varvec{w}}{^{\prime}}{\varvec{L}}{{\varvec{f}}}^{s}$$

where ${{\varvec{w}}}^{^{\prime}}$ is the transpose of the vector ${\varvec{w}}$ whose elements for the electricity sector in India are unity and are 0 for other sectors.

### Consumption-based health impacts

Referring to the work of Nagashima et al. (2017)^17^, the number of premature deaths in India due to PM_2.5_ emissions from the power generation sector associated with final demand in country *s* can be estimated using the following equation:

3 $${m}^{s}={\alpha {{\varvec{w}}}^{^{\prime}}{\varvec{L}}{\varvec{f}}}^{s}=\alpha {e}^{s}$$

where $\alpha$ represents the number of PM_2.5_-induced premature deaths in India per unit output from the power generation sector.

### Identifying critical supply chain paths

Next, we identify the environmentally important supply chain paths by evaluating the contribution made by the supply chain paths that link consumption and production to the consumption-based PM_2.5_ emissions obtained above. If we perform a series expansion of the Leontief inverse matrix in Eq. (1), we obtain ${\varvec{L}}={({\varvec{I}}-{\varvec{A}})}^{-1}={\varvec{I}}+{\varvec{A}}+{{\varvec{A}}}^{2}+{{\varvec{A}}}^{3}+\dots$. Then, from Eq. (3), we can estimate the health impacts in India induced by consumption in country $s$ as follows:

4 $${m}^{s}=\alpha {{\varvec{w}}}^{^{\prime}}{{\varvec{f}}}^{s}+\alpha {{\varvec{w}}}^{^{\prime}}{\varvec{A}}{{\varvec{f}}}^{s}+\alpha {{\varvec{w}}}^{^{\prime}}{{\varvec{A}}}^{2}{{\varvec{f}}}^{s}+\alpha {{\varvec{w}}}^{^{\prime}}{{\varvec{A}}}^{3}{{\varvec{f}}}^{s}+\dots .$$

Next, let us define $\alpha {{\varvec{w}}}^{^{\prime}}$ as ${{\varvec{q}}}^{^{\prime}}$, which represents premature deaths attributable to the emissions of primary and secondary particulate matter per unit output of the power generation sector of India, and the other vector elements as zeros. In this case, we can rewrite Eq. (4) as follows:

5 $${m}^{s}={{\varvec{q}}}^{^{\prime}}{{\varvec{f}}}^{s}+{{\varvec{q}}}^{^{\prime}}{\varvec{A}}{{\varvec{f}}}^{s}+{{\varvec{q}}}^{^{\prime}}{{\varvec{A}}}^{2}{{\varvec{f}}}^{s}+{{\varvec{q}}}^{^{\prime}}{{\varvec{A}}}^{3}{{\varvec{f}}}^{s}+\dots .$$

If we focus on India (*IND*) as the final demand country, then Eq. (5) can be expressed as ${m}^{IND}={{\varvec{q}}}^{^{\prime}}{{\varvec{f}}}^{IND}+{{\varvec{q}}}^{^{\prime}}{\varvec{A}}{{\varvec{f}}}^{IND}+{{\varvec{q}}}^{^{\prime}}{{\varvec{A}}}^{2}{{\varvec{f}}}^{IND}+{{\varvec{q}}}^{^{\prime}}{{\varvec{A}}}^{3}{{\varvec{f}}}^{IND}+\dots$, where the first term on the right-hand side denotes premature deaths in India associated with electricity directly consumed by final consumers in India, the second term denotes the premature deaths in India associated with the electricity that is directly required to meet final demand of goods and services in India, and the other terms represent premature deaths in India that are associated with electricity that is indirectly required to meet final demand of goods and services in India. Using this equation, we can estimate the consumption-based health impacts associated with the final demand of other countries.

The consumption-based health impacts in India can also be estimated by the following supply chain equation:

6 $${m}^{s}=\sum_{v=1}^{R}\sum_{j=1}^{N}{q}_{Elec}^{IND}({I}_{Elec,j}^{IND,v}+{a}_{Elec,j}^{IND,v}+({{\varvec{A}}}^{2}{)}_{Elec,j}^{IND,v}+\left({{\varvec{A}}}^{3}{)}_{Elec,j}^{IND,v}\cdots \right){f}_{j}^{vs}=\sum_{v=1}^{R}\sum_{j=1}^{N}{q}_{Elec}^{IND}({I}_{Elec,j}^{IND,v}+{a}_{Elec,j}^{IND,v} +\sum_{t=1}^{R}\sum_{k=1}^{N}{a}_{Elec,k}^{IND,t}{a}_{kj}^{tv}+\sum_{u=1}^{R}\sum_{t=1}^{R}\sum_{l=1}^{N}\sum_{k=1}^{N}{a}_{Elec.l}^{IND,u}{a}_{lk}^{ut}{a}_{kj}^{tv}+\cdots ){f}_{j}^{vs}={q}_{Elec}^{IND}{f}_{Elec}^{IND,s}+\sum_{v=1}^{R}\sum_{k=1}^{N}{q}_{Elec}^{IND}{a}_{Elec,j}^{IND,v}{f}_{j}^{vs}+\sum_{t=1}^{R}\sum_{k=1}^{N}\sum_{v=1}^{R}\sum_{j=1}^{N}{q}_{Elec}^{IND}{a}_{Elec,k}^{IND,t}{a}_{kj}^{tv}{f}_{j}^{vs}+\cdots $$

where ${q}_{Elec}^{IND}$ represents the premature deaths attributable to the emission of primary and secondary particulate matter per unit output of the power generation sector of India, while ${a}_{Elec,j}^{IND,v}$ is the intermediate input for the power generation sector of India that is necessary per unit of production of the product of industry *j* in country *v*. The second term on the right-hand side of Eq. (6), ${q}_{Elec}^{IND}{{a}_{Elec,j}^{IND,v}f}_{j}^{vs}$, expresses the health impacts in India due to PM_2.5_ emissions induced by the production of electricity in India that is required by industry *j* to produce the products in country *v* to satisfy final demand in country *s*. From the second term on the right-hand side of Eq. (6), we can identify the supply chain paths, such as “health impacts on India (impact receptor country)” → “electricity of India (emission source country)” → “industry *j* of country *v* (transmission country)” → “final demand in country *s* (i.e., consumption driver country).”

## Data and computation

We used EXIOBASE 3 data for 2010^23,24^. The EXIOBASE is a MRIO table that includes 44 countries, five international regions (Middle East, Other Asia, Other America, Other Europe, and Other Africa), and 163 industrial sectors. In this study, we constructed a “new electricity sector of India” by aggregating three power generation sectors in EXIOBASE 3 (i.e., “Production of electricity by coal,” “Production of electricity by petroleum and other oil derivatives,” and “Production of electricity by biomass and waste”) whose activities emit PM_2.5_^7^ to ensure that the input-out table corresponds to the emission inventory data.

This study considered 7985 sectors (48 countries and regions × 163 industrial sectors, and 161 industries in India) in our MRIO framework. Consequently, the number of supply chain paths in the 0th tier expressed by the first term on the right-hand side of Eq. (6) (i.e., ${q}_{Elec}^{IND}{f}_{Elec}^{IND,s}$) is 49, the number of paths in the first tier (i.e., $\sum\nolimits_{v = 1}^{R} {\sum\nolimits_{j = 1}^{N} {q_{Elec}^{IND} a_{Elec,j}^{IND,v} f_{j}^{vs} } }$) is $163\times 48+161=7985$, the number of paths in the second tier is $798{5}^{2}$, and the number in the *n*th tier is ${7985}^{n}$. However, we encountered a computation problem, as the number of computations for the total emissions in a given tier increased rapidly as the tier number increased. Therefore, for the purposes of this study, we set a threshold to disregard supply chain paths that (1) accounted for less than 0.001% of the total consumption-based health impacts for the paths triggered by Indian final demand, and (2) induced less than one premature death in the paths triggered by international final demand. As a result, we only considered paths up to the fourth tier, which includes more than 95% of consumption-based health impacts derived from the final demand in India. Considering international demand, we focused on the paths driven by final demand in the top three driver countries or regions, i.e., the Middle East, the USA, and China. In terms of consumption-based health impacts, our estimates covered more than 90% (Middle East), 85% (USA), and 60% (China) of the deaths induced in India. The Middle East region includes the following 14 countries: Bahrain, Egypt, Iran, Iraq, Israel, Jordan, Kuwait, Lebanon, Oman, Qatar, Saudi Arabia, Syria, the United Arab Emirates, and Yemen.

We also used data on PM_2.5_ emissions by power sector and Source Receptor Relationship (SRR) matrix estimated in a previous study^25^. In that study, the SRR matrix was estimated using an atmospheric transport model with a focus on Asian countries, including India. Modeling simulations were performed using the Community Multiscale Air Quality modeling system^26^, and the Weather Research and Forecasting (WRF) modeling technique^27^ was used to calculate meteorological field conditions. Further, the Emission Database for Global Atmospheric Research (EDGAR) inventory map^28^ was used to generate a 0.1° resolution map of sectoral emissions. The simulations were used to estimate the yearly averaged regional concentrations of primary PM_2.5_ (black carbon and organic carbon) and secondary precursors (NO_x_, SO_2_, NH_3_, CO, and NMVOC) emitted from all sectors and each individual sector in India, respectively. The grid consisted of 0.5° × 0.5° (about 45 km × 45 km) squares, with Papua New Guinea in the east, Iran to the west, Australia to the south, and Russia to the north. First, the human health effects (i.e., premature deaths) for each age group in each grid induced by the pollution concentrations from *all sectors* in India were estimated using the PM_2.5_ concentration map, an integrated exposure–response model^29,30^, and population distribution data by sex and age^31^. The relative risks of five diseases (ischemic heart disease, COPD, cerebrovascular disease, lung cancer, and LRI) were calculated for each age group using the integrated exposure–response model. The number of premature deaths in each grid was estimated using the same method as Apte et al. (2015). To calculate the contribution of PM_2.5_ emissions to the total premature deaths for each sector associated with PM_2.5_ emissions in India, we adopted a proportional attribution assumption, in which the health impacts were proportional to the PM_2.5_ concentration generated by each sector. We also employed the proportional assumption of PM_2.5_ concentration to both primary PM_2.5_ emissions and secondary precursors emissions to obtain $\alpha$ in Eqs. (3) and (4).

## Results

### Consumption-based health impact analysis

We first calculated the direct and indirect impacts of the power sector in India on the on the total number of PM_2.5_-related premature deaths. A direct health impact was defined as when electricity is consumed as a final product, mainly by households. An indirect impact was defined as when electricity is consumed as an intermediate product, mainly by industries that engage in production activities. The former can be calculated using ${q}_{Elec}^{IND}{f}_{Elec}^{IND,s}$, while the latter can be calculated by subtracting the direct health impact from the total of the consumption-based health impacts triggered by the final demand of countries, $\sum_{s=1}^{R}{m}^{s}$. The findings showed that the indirect impact was approximately four times larger than the direct impact. The findings also showed that it is important to further investigate the indirect impacts in order to better understand the mechanisms underlying mortality generation and to identify the stakeholders responsible, despite the complexity of the global supply chain.

**Figure 1:**
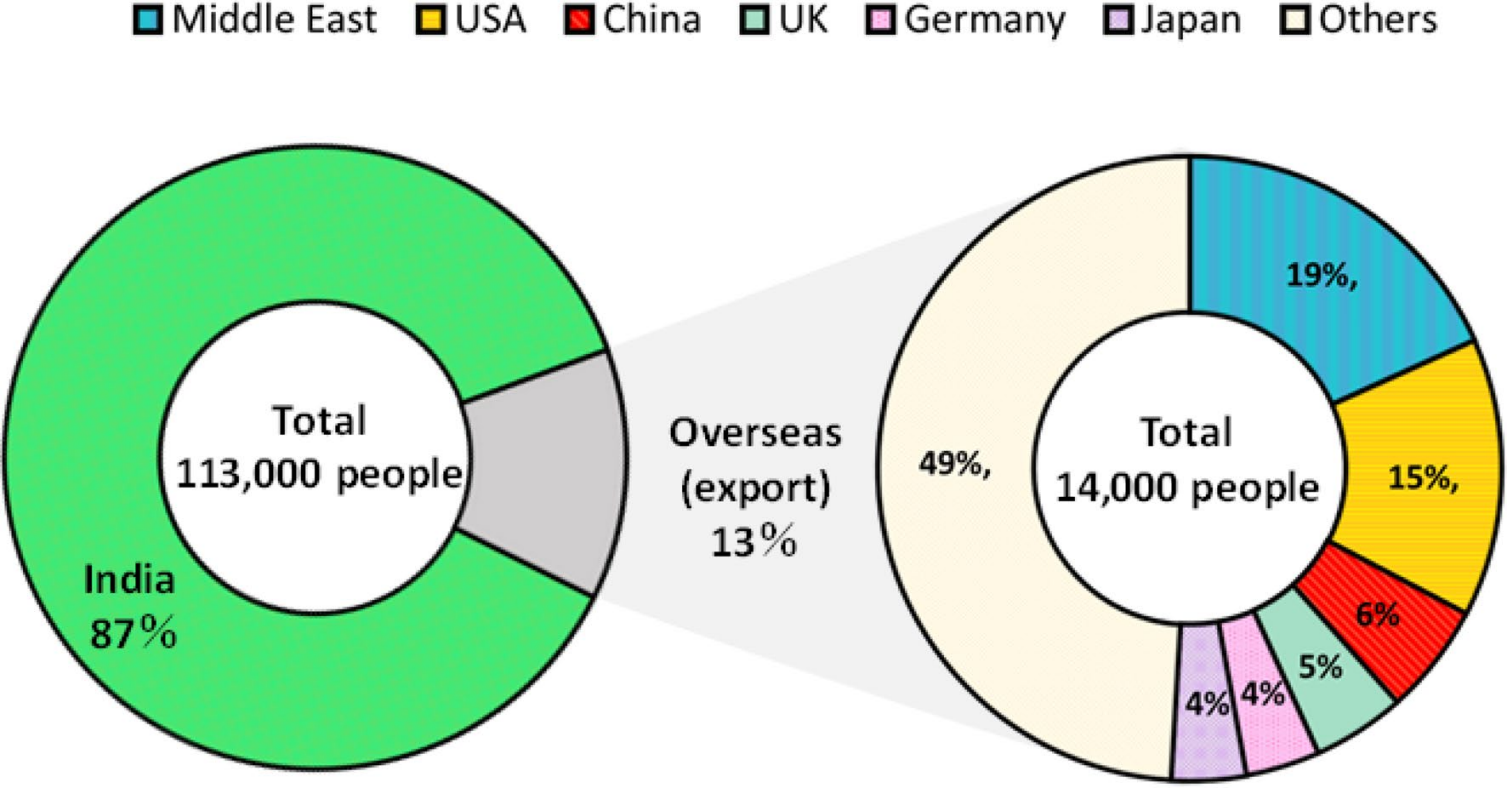
Consumption-based health impact in India triggered by domestic and international final demand.

Figure 1 shows the consumption-based health impacts (i.e., number of deaths) in India that are attributable to PM_2.5_ emissions induced by power generation in India for domestic and foreign final demand. The consumption-based mortality in India estimated in this study is 113,000 people in 2010. It is important to note that the consumption-based health impacts in India induced by the final demand of countries ($\sum_{s=1}^{R}{m}^{s}$) is the same as the production-based health impacts. Guttikunda and Jawahar (2014)^12^ reported that the production-based mortality induced by coal-fired power generation in India in the same year was between 80,000 and 115,000 people. Although the estimation methodology is different, the results of this study are consistent with those of Guttikunda and Jawahar (2014)^12^.

Importantly, 87% of air pollution-related deaths in India were due to the domestic final demand of India, whereas 13% was due to the final demand of other countries (Fig. 1). The Middle East was the largest contributor to these health impacts, accounting for 19% of the total impact, followed by the USA, China, and the United Kingdom, which contributed 15%, 6%, and 5%, respectively. We note that both domestic and foreign drivers account for health impacts in India via direct and indirect electricity use.

Next, we applied the SPA to 163 domestic sectors and the three largest contributors to final demand (i.e., the Middle East, the USA, and China), as well as India, to identify critical supply chain paths and to understand how these final demands are linked to the health impact of PM_2.5_ emissions from the power generation sector in India.

### Health impact analysis based on India’s final demand

**Figure 2:**
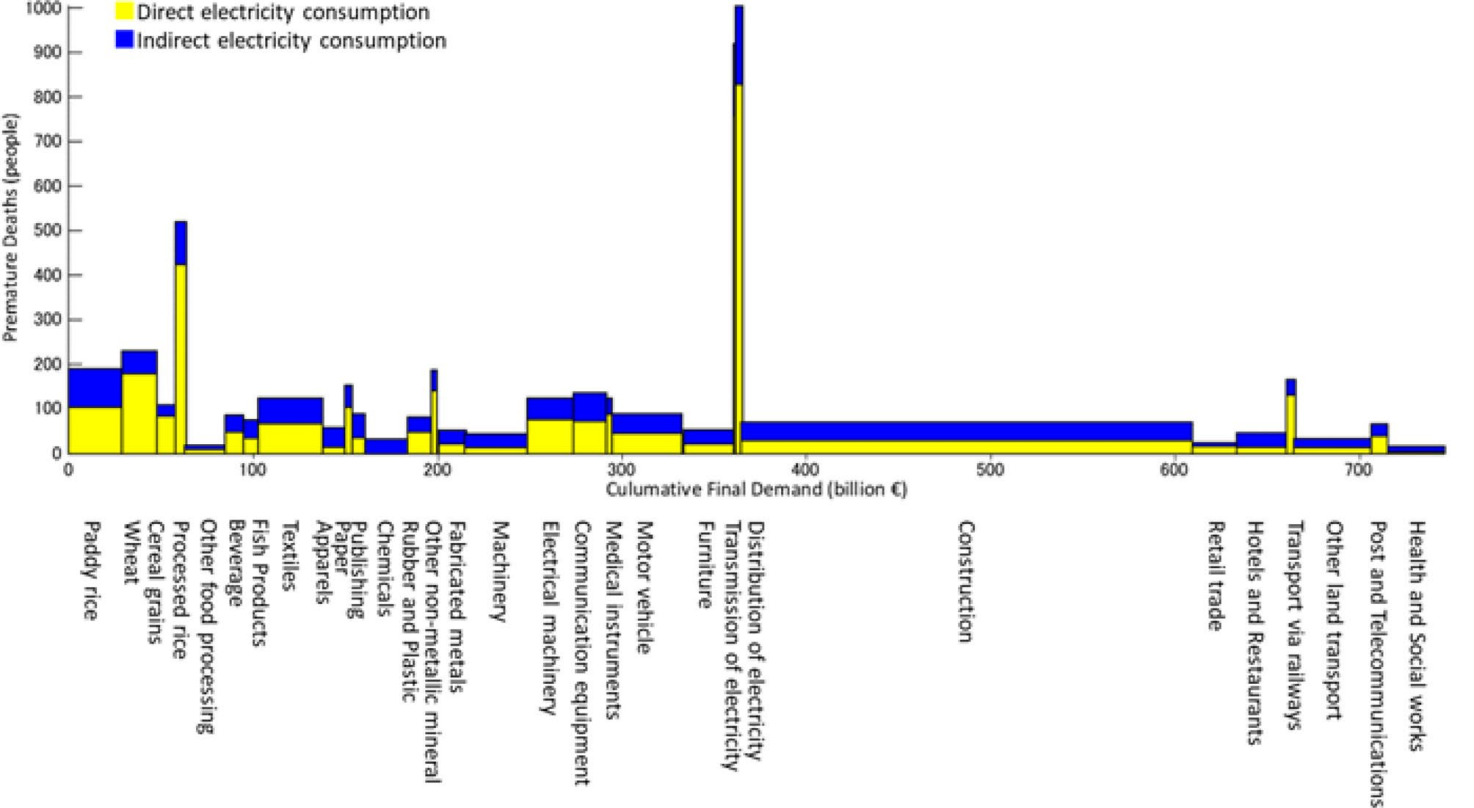
Contribution of electricity consumption by India’s domestic sectors to health impacts.

Figure 2 shows the contribution of final demand sectors in India to PM_2.5_ emission-related mortality in India. The rectangular areas in Fig. 2, calculated by multiplying the size of a certain final demand sector’s final demand (*x*-axis: along the bottom) by the number of premature deaths per unit of that sector’s final demand (*y*-axis: height), represents the number of premature deaths caused by that sector through direct and indirect electricity consumption. The *x*- and *y*-axes can also be thought of as “market scale” and “intensity of electricity consumption”, respectively.

According to Fig. 2, the construction sector is responsible for 17,500 PM_2.5_ emission-related premature deaths, the greatest number of all final sectors (Table 1). As the construction sector’s rectangular area is characterized by having a considerably wider base compared to its height, we can see that the market scale of the construction sector—which reflects India’s active domestic infrastructural development—contributes greatly to the number of premature deaths. In contrast, the paddy rice sector is responsible for 5500 premature deaths and is the second largest contributor in the country (Table 1). While the construction sector’s rectangle has a base that is wider than its height, the paddy rice sector’s rectangle is taller than it is wide; therefore, it is the intensity of its electricity consumption that contributes greatly to premature deaths. Consequently, reducing the premature deaths ascribed to the paddy rice sector will require a considerable reduction of its electricity consumption through basic policies, such as capital investment and re-examining systems. This is a characteristic shared with other agricultural sectors, such as the wheat and processed rice sectors.

**Table 1:** Highest PM_2.5_-related health impacts induced by electricity consumption in domestic final demand sectors in India.

Final demand sector premature	Deaths (*n*)
Construction	17,500
Paddy rice	5500
Textiles	4370
Wheat	4360
Distribution and trade of electricity	3800
Motor vehicle	3400
Electrical machinery	3050
Processed rice	3000
Communication equipment	2500

The textile sector is the third-largest contributor to premature deaths in India, being responsible for 4370 deaths (Table 1). Unlike the rectangles of the construction and rice paddy sectors mentioned above, the rectangle of the textile sector is close to a perfect square and, as such, is a sector in which market scale and intensity of electricity consumption both contribute equally to premature deaths.

Considering the proportions of the upper and lower rectangles shown in Fig. 2, we can infer the proportion of (1) premature deaths associated with the electricity required to satisfy each sector’s final demand (*direct electricity consumption*) in relation to (2) premature deaths associated with the electricity required to produce the intermediate goods necessary to satisfy each sector’s final demand (*indirect electricity consumption*). The former tends to be greater than the latter in the case of paddy rice, wheat, and cereal grains in the agricultural sector, and electrical machinery and communication equipment in the manufacturing sector. These sectors have a large number of premature deaths and the supply chain paths that involve them are: (1) Electrical machinery (IND) → Communication Equipment (IND) → Electricity (IND) (50 premature deaths); (2) Electrical machinery (IND) → Fabricated metal products (IND) → Electricity (IND) (29 premature deaths); (3) Communication equipment (IND) → Electrical machinery (IND) → Electricity (IND) (211 premature deaths); and (4) Communication equipment (IND) → Post and telecommunications → Electricity (IND) (70 premature deaths).

In contrast, in the service sector—such as hotels and restaurants and other land transport—more premature deaths are due to indirect than direct electricity consumption. The key path for hotels and restaurants is: Hotels and Restaurants (IND) → Paddy rice (IND) → Electricity (IND) (89 premature deaths) and Hotels and Restaurants (IND) → Wheat (IND) → Electricity (IND) (70 premature deaths). In the case of *other land transport*, the key path is: Other land transport (IND) → Rubber and Plastic products (IND) → Electricity (IND) (74 premature deaths) and Other land transport (IND) → Textiles (IND) → Electricity (IND) (45 premature deaths).

As mentioned above, the *construction* sector was the final demand sector with the highest number of PM_2.5_ emission-related premature deaths, and the number of premature deaths induced by *direct electricity consumption* was roughly the same as those by *indirect electricity consumption* (Fig. 2). The key paths involving these sectors were: Construction (IND) → Cement, Lime, and Plaster (IND) → Electricity (IND) (1634 premature deaths); Construction (IND) → Ceramic goods (IND) → Electricity (IND) (1016 premature deaths). From these results, it is clear that even if a particular final demand sector’s electricity demand (the height in Fig. 2) is low, that sector still has an indirect, but marked impact on the number of premature deaths. This impact occurs through Scope 3 PM_2.5_ emissions, which are caused by investing the goods produced in these sectors with intense electricity demand into intermediate goods. To efficiently reduce the health impact of PM_2.5_ emissions caused by the power sector, policies to reduce electricity consumption should focus on the intermediate goods sectors that provide a given final demand sector with intermediate goods, as well as the final demand sector itself.

### Analysis of health impacts triggered by foreign final demand

Next, we present the results of the SPA to break down the consumption-based health impacts triggered by foreign final demand for different global supply chain paths extending from final demand to producers.

**Figure 3:**
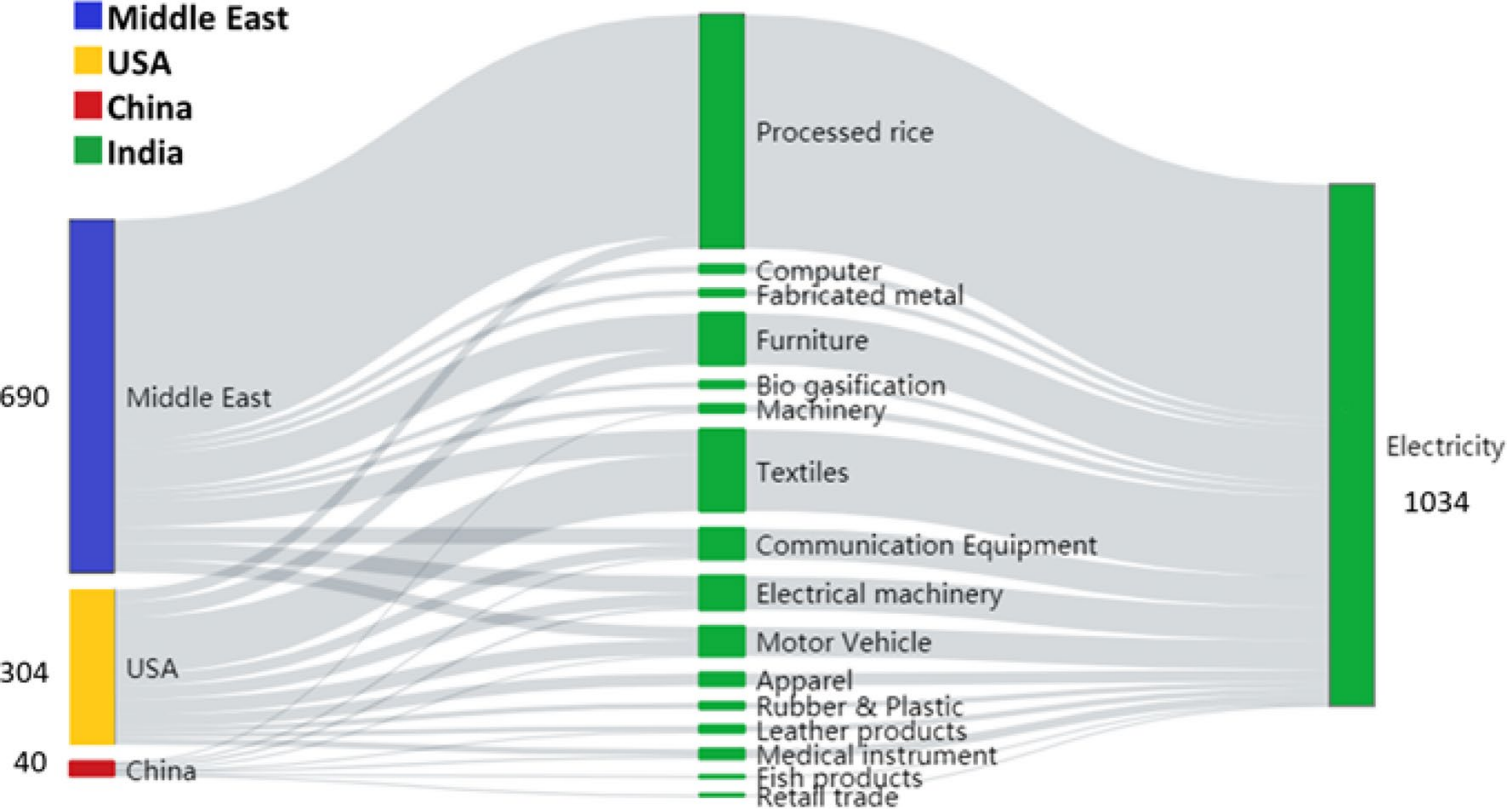
Top 10 supply chain paths ranked in terms of health impact driven by the Middle East, USA, China in the first tier.

**Figure 4:**
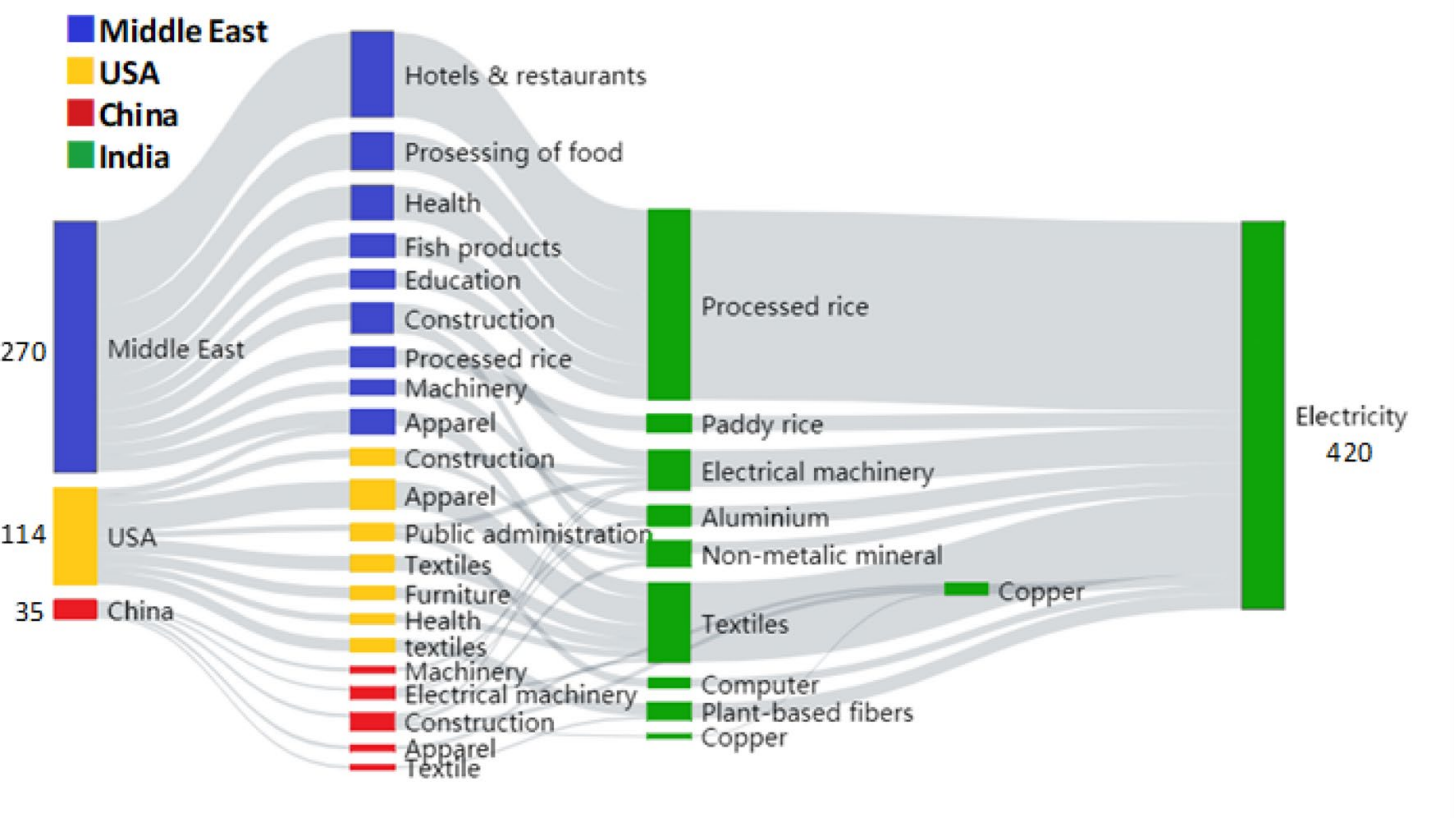
Top 10 supply chain paths ranked in terms of health impact driven by the Middle East, USA, China in the second to fourth tiers.

Figure 3 shows a Sankey Diagram composed of the top 10 supply chain paths in tier 1 driven by final demands in the Middle East, the USA, and China (i.e., a total of 30 paths). The path with the largest health impact was: Middle East → Processed rice (IND) → Electricity (IND), which induced 433 premature deaths. Middle Eastern countries import processed rice produced in India; consequently, rice processing induces a high health impact through PM_2.5_ emissions from power plants. Based on imports of processed rice, Middle Eastern countries have a large responsibility concerning India’s premature death rate. Figure 4 also shows the path USA → Textiles (IND) → Electricity (IND), which also has a high health impact, inducing 110 premature deaths. The USA imports textiles produced in India, and the textiles sector in India induces a health impact in India via electricity consumption. We therefore posit that the USA should also be involved in mitigation measures related to imports of Indian textiles.

Figure 4 is a Sankey Diagram showing the top 10 supply chain paths in tiers 2 to 4 that are driven by final demand sectors in the Middle East, the USA, and China (Total 30 paths). The figure shows that there are high-ranking paths centered on imports of processed rice in India via food processing and service sectors in the Middle East: Middle East → Hotels and Restaurants (Middle East) → Processed rice (IND) → Electricity (IND) (80 premature deaths); Middle East → Processing of food (Middle East) → Processed rice (IND) → Electricity (IND) (38 premature deaths); and Middle East → Health and social works (Middle East) → Electricity (IND) (35 premature deaths). The textile supply chains linking India with the USA also have a high health impact: USA → Apparel (USA) → Textiles (IND) → Electricity (IND) (28 premature deaths); USA → Textiles (USA) → Textiles (IND) → Electricity (IND) (15 premature deaths); and USA → Textiles (IND) → Plant-based fiber (IND) → Electricity (IND) (13 premature deaths).

## Discussion and conclusion

We first calculated the consumption-based health impact associated with PM_2.5_ emissions from the domestic power sector in India by combining a health impact database with a global MRIO database. We then used an SPA to reveal how the PM_2.5_ emitter (Electricity sector in India) is linked to domestic Indian final demand and international demand in a global supply chain. The results showed that the total number of premature deaths attributable to PM_2.5_ emissions from the power sector was 113,000 and that 87% of those deaths was induced by Indian final demand and 13% was induced by international final demand (i.e., exports from India). Construction was the largest domestic contributor to PM_2.5_ emission-related mortality in India as it consumed the most electricity, either directly or indirectly.

A key finding of this study was that supply chain paths centered around agriculture, e.g., Paddy rice (IND) → Electricity (IND), Processed rice (IND) → Electricity (IND), Middle East (Final demand) → Processed rice (IND) → Electricity (IND), played a major role in premature deaths associated with PM_2.5_ emissions from the power sector in India. Although it is well-known that agricultural sectors in India, such as paddy rice cultivation, directly emit considerable quantities of PM_2.5_ by burning fields^32^, we found that indirect emissions via its electricity consumption also considerably affected human health in India.

This finding also showed that electricity consumption at the cultivation stage of paddy rice and wheat was responsible for approximately 5500 and 4360 PM_2.5_ emission-related premature deaths, respectively (Table 1). One of the primary causes of electricity consumption in the paddy rice and wheat sector is electric pumps for irrigation, and India has installed more than 20 million pump sets^33^. Rice and wheat are also both highly water-intensive crops and it has been shown that irrigation agriculture in India is structured in a way that consumes more electricity than is necessary^34^.

The Indian government has set a low fixed price for electricity for agricultural use through subsidies, and under this system, irrigation farmers have no incentive to conserve electricity. The Indian government should immediately increase the price of electricity for agricultural use and abolish the subsidies for the agricultural industry. Instead, the government should subsidize capital investment for irrigation farmers, e.g., by investing in energy-saving irrigation pumps, to move the agricultural sector in a more energy-saving direction.

As India is a vast country, there is considerable regional variation in economic activities such as agriculture. Although MRIO analysis of sub-regions in India can provide detailed information on the spatial heterogeneity in production and consumption (e.g., Nagashima, 2018^35^), there is, at present, no MRIO database available for Indian sub-regions. As a result, we used the following simplified sub-regional analysis^36^ to spatially break down the domestic supply chain paths in India. We calculated the contributions of states and union territories of India to the PM_2.5_ emission-related premature deaths along the supply-chains of rice (wheat) by distributing the total number of premature deaths induced by electricity consumption required for rice (wheat) cultivation according to the share of rice (wheat) production in each state and union territory (Table S1).

**Figure 5:**
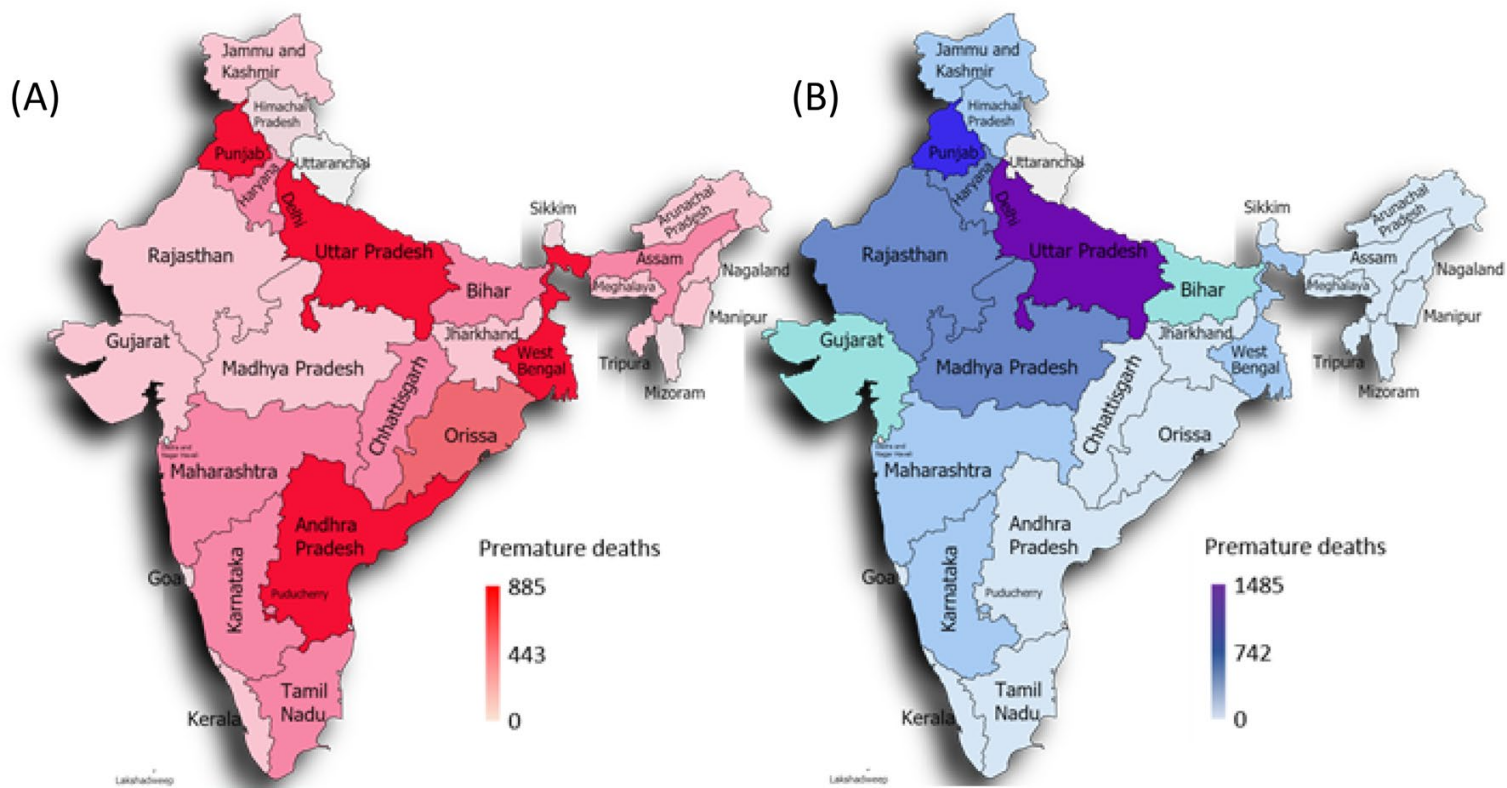
Spatial distributions of the PM_2.5_ emission-related premature deaths via rice (**A**) and wheat (**B**) supply chains. Maps generated in Paintmaps.com (https://paintmaps.com/).

Figure 5A,B show the spatial distributions of the PM_2.5_ emission-related premature deaths in the rice and wheat supply chains, respectively. The results show that the three states of West Bengal, Uttar Pradesh, and Punjab accounted for approximately 40% of the premature deaths that are attributable to rice supply, while the three states of Uttar Pradesh, Punjab, and Haryana accounted for 66% of the premature deaths attributable to wheat supply. We suggest that the local governments of West Bengal, Uttar Pradesh, Punjab, and Haryana should implement aggressive electricity-saving policies in the areas of rice and wheat production in order to mitigate against PM_2.5_ emissions from the power sector in India.

The processed rice sector was responsible for approximately 3000 premature deaths due to the power required for production to meet domestic demand, and approximately 600 premature deaths for production to meet the export demand of the Middle East. Rice processing can be broadly divided into four processes—parboiling to remove pesticides and insects, de-husking, selection, and preservation—and this sector requires large amounts of electricity due to the increasing mechanization required to polish such large quantities of rice^37^. In addition, the rice processing equipment in India is old, and while the Indian government is supporting the introduction of newer and more energy-efficient equipment, its penetration is only 50%^38^. One reason for this low penetration is the high installation costs associated with this equipment. The second reason is that, even if the government were to encourage technical improvements in the rice processing sector through subsidies and regulations, ensuring technical improvements and maintaining efficiency would incur high monitoring costs for the government in a developing country such as India. To avoid this, it is crucial to motivate the rice processing sector itself to take the initiative for conducting technical improvements. Considering the key supply chain paths described in this study that involve India’s rice processing sector, “environmental labeling” is very important; that is, communicating the number of premature deaths (environmental data) caused by the production of rice when that rice is sold to consumers in India and the Middle East. This environmental labeling policy would allow India’s rice processing sector to introduce and use efficient technologies of its own initiative to improve environmental data.

The results of this study showed that, at the consumption stage of rice, a significant proportion of electricity-induced premature deaths are caused by the importation of rice by the Middle East. In 2017, India was the world’s largest rice exporter^39^ and countries in the Middle East (Iran, Saudi Arabia, and Iraq) were the top three destinations for its rice^40^. While Middle Eastern countries import a large amount of rice from India, Saudi Arabia ranked first in the world for food wastage per capita in 2017, including rice^41^. Purchasing more Indian rice than is necessary only to waste it is a consumption pattern that results in additional and unnecessary domestic electricity consumption in India, which, in turn, leads to an increase in health risks due to the increase in PM_2.5_ emissions during electricity generation. Suppressing this wasteful consumption pattern will require measures such as levying a pollution tax on imports of rice produced in India and educating consumers about the effects of indirect pollution beyond their borders.

As India’s population is expected to increase further in the future, rice and other food sectors are industries that not only generate value added, but are also essential for feeding one of the largest populations in the world. With increasing the food demand, higher mechanization of agriculture will increase electricity consumption in India. To mitigate health burden triggered by the agricultural production, improving energy efficiency in these sectors is crucial for sustainable agricultural production. It is important to reduce the electricity consumption through introducing equipment with higher energy efficiency as well as strengthening monitoring system of measuring potential energy savings associated with pumping water for irrigation^42^. Ensuring the sustainable production in these sectors is a pressing challenge and an issue that must be prioritized by all stakeholders.

The limitations of this study are the following.

1. It should be noted that our sub-regional results described in Fig. 5 are based on the simplified spatial attribution analysis. Multi-regional input–output analysis with a focus of the Indian economy could elaborate the sub-regional results. This elaboration is an important follow-up study.
2. This study focused only on PM_2.5_ emissions, therefore we did not consider health burdens triggered by other pollutants. The analysis scope should be expanded in a future study.
3. The share of unorganized sector (i.e., informal sector) in net domestic product in India was 57% in 2002^43^, and thus the informal sector played a crucial role in the Indian economy. It is important to note that the social accounting matrix for India was estimated by using the shares of net domestic product or factor incomes for the unorganized and organized enterprises^43^. The net domestic product for the informal sector was estimated based on detailed employment surveys in India^43^. As a result, the input–output table for India included in the EXIOBASE3 captures economic activities in both formal and informal sectors. Therefore, we implicitly considered the PM_2.5_ emissions from the informal sector. The emission results largely depend on the System of National Accounts of India. The detailed disaggregation analysis with a focus of the informal sector is also a future study.

## Supplementary Information

Supplementary Information.
