# Supplementary material for: Critical supply chains for mitigating PM2.5 emission-related mortalities in India
Source: Sci Rep. 2021 Jun 7;11:11914. doi: 10.1038/s41598-021-91438-2 (PMC8185009; doi:10.1038/s41598-021-91438-2)
Supplement: Supplementary file 1 — Supplementary Information. [file 41598_2021_91438_MOESM1_ESM.docx]

**Critical Supply Chains for Mitigating PM_2.5_ Emission-related Mortalities in India**

*by*

Haruka MITOMA^a*^, Fumiya NAGASHIMA^b^, Shigemi KAGAWA^c^ and

Keisuke NANSAI^d,e^

*Correspondence to [mitoma.haruka.648@s.kyushu-u.ac.jp](mailto:mitoma.haruka.648@s.kyushu-u.ac.jpm)

^a^ Graduate School of Economics, Kyushu University, Fukuoka, Japan

^b^ Faculty of Economics, Kindai University, Osaka, Japan

^c^ Faculty of Economics, Kyushu University, Fukuoka, Japan

^d^ National Institute for Environmental Studies, Ibaraki, Japan

^e^ ISA, School of Physics, The University of Sydney, NSW, Australia

**Supplementary Information**

**Table S1:** State-wise production of rice and wheat in 2010

-: Not Available.

Source: Reserve Bank of India (2021) Handbook of Statistics on Indian States, webpage

<https://rbi.org.in/Scripts/AnnualPublications.aspx%3Fhead%3DHandbook%20of%20Statistics%20on%20Indian%20States> (accessed March 28, 2021)
